# Comprehensive constitutive modeling and analysis of multi-elastic polydimethylsiloxane (PDMS) for wearable device simulations

**DOI:** 10.1038/s41598-023-45372-0

**Published:** 2023-10-27

**Authors:** Nora Asyikin Zulkifli, Geon Dae Moon, Dong Choon Hyun, Sungwon Lee

**Affiliations:** 1 Department of Physics and Chemistry, Daegu Gyeongbuk Institute of Science and Technology (DGIST), Dalseong-Gun, Daegu, 42988 Republic of Korea; 2 Dongnam Division, Korea Institute of Industrial Technology, Busan, 46938 Republic of Korea; 3 Department of Polymer Science and Engineering, Kyungpook National University (KNU), Daegu, 41566 Republic of Korea

**Keywords:** Electronic devices, Polymers, Computational methods, Mechanical properties

## Abstract

Within the field of wearable devices, polydimethylsiloxane (PDMS) has long been one of the most prominent materials utilized. It is therefore unsurprising that demands for its usage has now extended beyond experimental works into computational simulations, particularly those involving finite element method (FEM). To replicate the mechanical properties of PDMS in FEM, an accurate constitutive model is required, preferably one that encompasses wide ranges of PDMS elasticity. In this study, we determine Mooney–Rivlin 5 parameters as the best hyperelastic model fitted against PDMS experimental data, and proceed to construct a parameter correlation plot combining PDMS of different elasticities together. Experimental validation using PDMS samples fabricated via 3D-printed molds is then performed using parameters extracted from this plot, showing good agreement between simulation and experimental result. In addition, to reflect model applicability, simulations related to basic mechanical deformations involved in flexible devices (compression, stretching, bending and twisting) are performed and analyzed. Further analysis is also performed to investigate the effect of combining different experimental datasets as input into the model. We expect our work to be potentially helpful to be applied as both framework and database for wearable device engineers and researchers who are experimenting with varying PDMS concentrations and modulus.

## Introduction

The wearable electronics field has always been concerned with the proper integration of external electronics systems with biological structures. The idealized concept of such an integration takes form in a vision of thin, stretchable devices, seamlessly incorporated into or onto our bodies. Such devices require utilization of incredibly flexible and stretchable inert materials as the main substrate or encapsulating body. While new materials ranging from rubber, plastics and textiles are often introduced and integrated with electronics to fulfil this goal, many researchers still opt for one of the earliest, classic materials used in this field; the silicon-based elastomer, polydimethylsiloxane (PDMS).

PDMS’s wide applicability can be contributed to its non-toxic and biocompatible attributes, as well as its ease in procurement and handling. But at its most fundamental level, PDMS is widely used due to its mechanical properties, namely its robustness and high degree of tunability. As an elastomer, it possesses a higher degree of elasticity and viscoelasticity while still retaining many polymer-like properties. It is not a surprise then, that PDMS is a common material used as substrates in many mechanical related flexible devices such as strain sensors and pressure sensors^1,2^.

Beyond the scope of experimental work, researchers in wearable electronics typically utilize tools involving numerical and computational analysis such as the finite element method (FEM) to perform investigations that may be limited in real life due to physical or time constraints. Within various complicated FEM simulations, PDMS may not play a major active role in most analysis, but since it is so widely used in wearable device design, modeling PDMS as a material becomes a necessity. To strengthen the accuracy of an FEM simulation involving PDMS, the model representing PDMS as a material should be accurate and similar to its real-life counterpart. But to enable fast and convenient access to large arrays of PDMS tunable properties, the model should also ideally be comprehensive and encompasses a broad range of potential roles.

While several papers have attempted to model PDMS in the past, a wide range of results can be obtained depending on the uses of the PDMS. Examples include PDMS for microfluidic device applications^3^, as channels mimicking blood vessels^4^, and as coating on micropillars^5^. The closest study carried out for wearable device uses would be a model for wrinkling and restabilization analyses of PDMS membrane^6^, which is a common behavior observed in materials used in wearable devices. Other studies on PDMS modeling without any emphasis on applications were mostly carried out on more specific scopes, such as in very small deformations caused by water droplets^7^, and using data for large shear strain deformations^8^. In our paper, we would like to focus on a more generic but comprehensive modeling, specific towards the fundamental mechanical deformations observed in wearable device uses, such as stretching, compression, bending and twisting.

To illustrate the applicability of the model, we varied the Elastic Modulus of the representative PDMS samples. This has already been carried out in a simple investigation by Kim et al.^9^, but while the paper did report singular data on a variety of PDMS modulus, it did not make any further elaborations or analyses, nor did they attempt to correlate their datasets to each other. On the other hand, we constructed a material parameter correlation plot which will allow users to easily determine parameters required in FEM for a wide range of PDMS concentrations without having to conduct any extra experiments. This has only been done in a research studying constitutive modeling of hydrogels^10^, but has never been attempted for PDMS. In addition, as an alternative option in the event of calculation errors or non-convergence, we showed comparisons between different combinations of experimental datasets depending on its usage within a structural deformation study.

**Figure 1:**
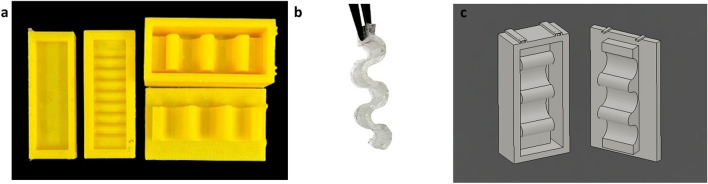
(**a**) 3D-printed PDMS molds and scaffolds for experimental validation samples. (**b, c)** PDMS taking the form of a symmetrical ribbon structure: (**b**) PDMS sample obtained from the scaffolds; (**c**) Computer-aided design (CAD) of the 3D-printed PLA scaffolds drawn via Autodesk Fusion 360 software.

Finally, in an attempt to validate our simulations, comparisons were performed with PDMS samples fabricated from 3D-printed polylactic acid (PLA) molds and scaffolds (Fig. 1a). One of the resulting PDMS designs took the form of a symmetrical ribbon structure similar to the well known buckled nanoribbon introduced in the pioneer research by Sun et al.^11^ (Fig. 1b, c). This was an unprecedented structural design in the realm of PDMS fabrication, where structures are typically asymmetrical due to difficulties in controlling the viscosity of pre-cured PDMS.

## Results and discussion

### Stress–strain characterizations of PDMS samples

**Figure 2:**
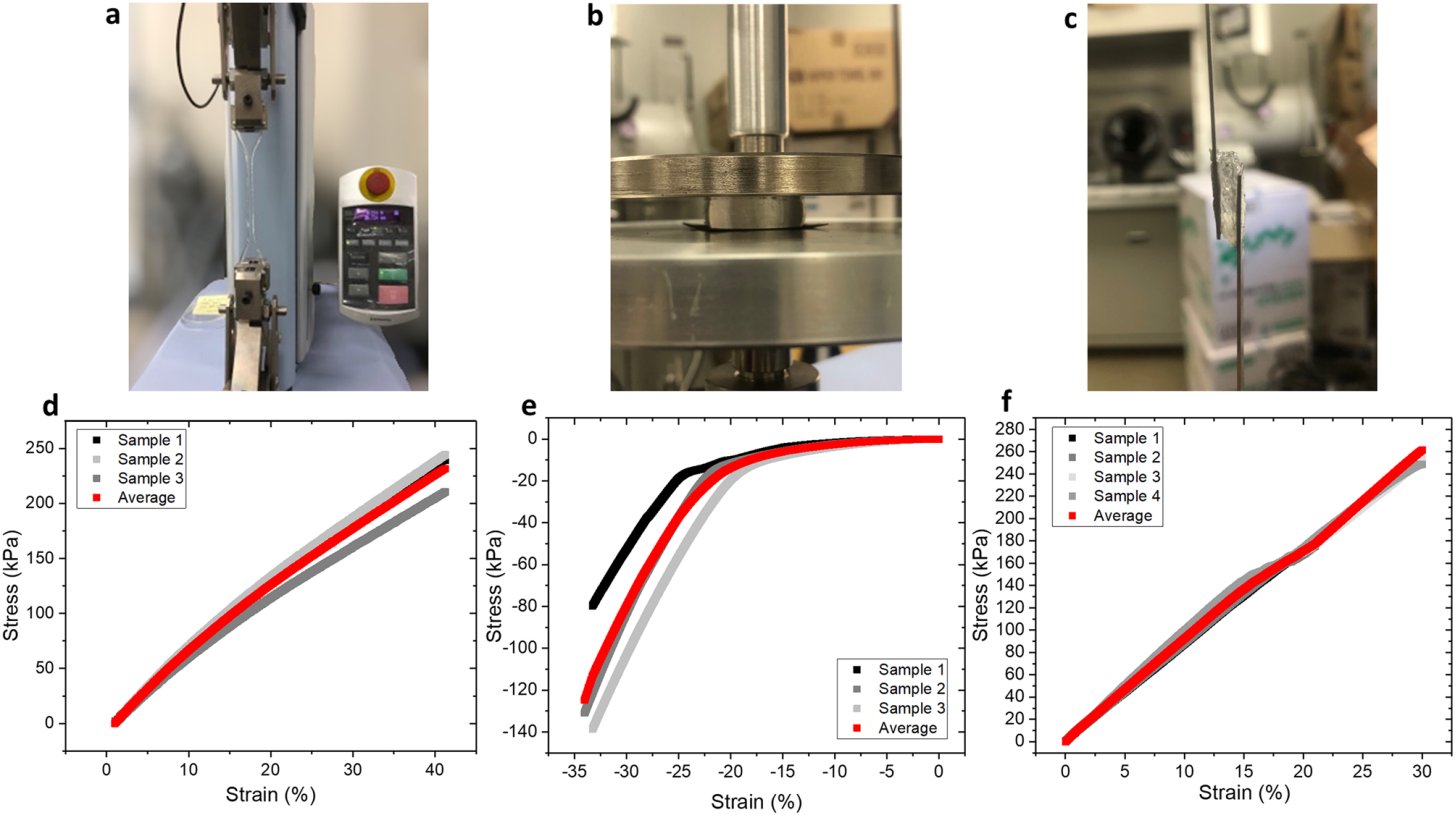
(**a**–**c)** Mechanical deformation tests performed on PDMS sample, *n* = 0.08: (**a**) uniaxial tensile test; (**b**) uniaxial compressive test; (**c**) simple shear test. (**d**–**f)** Corresponding nominal stress–strain plots for: (**d**) uniaxial tensile test; (**e**) uniaxial compressive test; (**f**) simple shear test.

In the first part of the study, three mechanical experiments were conducted on PDMS samples: uniaxial tensile test (Fig. 2a), uniaxial compressive test (Fig. 2b) and planar shear test (Fig. 2c). All samples were fabricated based on the standards outlined by ASTM International. Details on each sample’s dimensions are compiled in Supplementary Information 1. To vary the elasticity of the PDMS, the base to curing agent ratio was changed proportionately. A variable, *n* was introduced as follows:

1 $$ n = \frac{{curing \,agent \left( {wt\% } \right)}}{{base \left( {wt\% } \right)}} $$

However, in this initial stage, only PDMS of *n* = 0.08 were fabricated and tested. The resulting nominal stress–strain plots for all three mechanical tests are shown in Fig. 2d–f. The red curve in each plot represents the average stress–strain plot calculated from all measured sets of data.

### Modeling of hyperelastic materials

To integrate data from the mechanical experiments into finite element analysis, a curve fitting technique was employed via the COMSOL Multiphysics software. This method allows characterization of the volumetric deformation of the samples through parameter estimation when the experimental data are fitted with theoretical material models. For simplification purposes, PDMS is assumed to be incompressible, which allows it to be fitted to hyperelastic material models. This approximation of incompressibility is a common assumption taken for most elastomer and rubber-like materials, due to their high Poisson’s ratio, of which a higher absolute value would usually dictate high incompressibility. In this case, PDMS has been reported to have a Poisson’s ratio of around 0.5, which is the maximum value for incompressibility. Throughout the years, many researchers have attempted to re-measure pristine PDMS’s Poisson’s ratio. As a consequence, while many novel measuring methodologies have been introduced, all results are still within the 1% margin of error from the original 0.5 value^12–14^. Thus, PDMS continues to be represented in models and simulations to be incompressible.

In the past, hyperelastic models have been used multiple times to model various rubber and elastomer specimens. For large deformations, these models are often expressed by elaborating strain energy density function, *W*, in the form of stretch and deformation tensor invariants, respectively^15,16^.

Stretch is typically defined as;

2 $$ \lambda = \frac{L}{{L_{0} }} $$

where $L$ is the length of the PDMS samples after deformation, and $L_{0}$ is the original length.

Since this study involves three deformation experiments (uniaxial tensile, uniaxial compression and simple shear), the invariants of deformation tensor, *I,* can be expressed as follows;

Uniaxial tensile:

3 $$ I_{1} = \lambda^{2} + \frac{2}{\lambda } $$

4 $$ I_{2} = 2\lambda + \frac{1}{{\lambda^{2} }} $$

Uniaxial compression:

5 $$ I_{1} = 2\lambda^{2} + \frac{1}{\lambda } $$

6 $$ I_{2} = \lambda^{4} + \frac{2}{{\lambda^{2} }} $$

Simple shear:

7 $$ I_{1} = I_{2} = \left( {\lambda - \frac{1}{\lambda }} \right)^{2} + 3 $$

Among the large variety of hyperelastic models available, this study focuses on five of them, chosen based on suitability to PDMS’s structural properties and ease of modeling. Multiple research on PDMS modeling have been conducted using these five hyperelastic models, with varying results depending on their methodologies and applicability^5,9^. Each of this model can be further elaborated to express the relationship between stress and strain of the material.

#### Neo-Hookean model

This model is typically used to represent the nonlinear behavior of some plastic and rubber-like materials under large deformation. As can be deduced from its name, this model is an adaptation of Hooke’s law and perfect elasticity is usually assumed at all stages of deformation^17^.

8 $$ W = \frac{\mu }{2}\left( {I_{1} - 3} \right) + \frac{1}{D}\left( {J - 1} \right)^{2} $$

where $\mu$ is the initial shear modulus of materials, $D$ is the material incompressibility constant, and *J* is the determinant of the elastic deformation gradient, *F*.

#### Mooney–Rivlin 2 parameters model

An improvement from the Neo-Hookean model, this model is typically depicted as a polynomial curve, ideal for representing rubbers and elastomers within a medium-to-large deformation range^18,19^.

9 $$ W = C_{10} \left( {I_{1} - 3} \right) + C_{01} \left( {I_{2} - 3} \right) + \frac{1}{D}\left( {J - 1} \right)^{2} $$

where $C_{10}$ and $C_{01}$ are Mooney–Rivlin material constants.

#### Mooney–Rivlin 5 parameter model

A higher number of order reflects the complexity of the constitutive relations between the model and the material, as well as increases its behavior of nonlinearity^18,19^.

10 $$ W = C_{10} \left( {I_{1} - 3} \right) + C_{01} \left( {I_{2} - 3} \right) + C_{20} \left( {I_{1} - 3} \right)^{2} + C_{11} \left( {I_{1} - 3} \right)\left( {I_{2} - 3} \right) + C_{02} \left( {I_{2} - 3} \right)^{2} + \frac{1}{D}\left( {J - 1} \right)^{2} $$

where $C_{10}$, $C_{01}$, $C_{20}$, $C_{11}$ and $C_{02}$ are Mooney–Rivlin material constants.

#### Ogden model

This is a relatively simple, versatile constitutive model for various rubber-like materials, polymer and even biological tissues^20^. The range of strain deformation applicable is incredibly high when compared with previously stated hyperelastic models.

11 $$ W = \mathop \sum \limits_{p = 1}^{N} \frac{{\mu_{p} }}{{\alpha_{p} }}\left( {\lambda_{1}^{{\alpha_{p} }} + \lambda_{2}^{{\alpha_{p} }} + \lambda_{3}^{{\alpha_{p} }} - 3} \right) + \mathop \sum \limits_{k = 1}^{N} \frac{1}{{D_{k} }}\left( {J - 1} \right)^{2k} $$

where $\mu_{p}$ and $\alpha_{p}$ are material constants for Ogden model, and *p* represents the number of Ogden terms used.

#### Yeoh model

Yeoh model is an improvisation of the Mooney–Rivlin model to fit materials with more rubber-like behaviors.^21^

12 $$ W = \mathop \sum \limits_{p = 1}^{N} c_{p} \left( {I_{1} - 3} \right)^{P} + \mathop \sum \limits_{k = 1}^{N} \frac{1}{{D_{k} }}\left( {J - 1} \right)^{2k} $$

where $c_{p}$ is Yeoh model’s material constant, and *p* represents the number of order used for Yeoh’s model.

### Selection of material models

In order to choose an optimized, accurate material model, a number of steps and considerations should generally be followed, although one may choose their own execution method and solver type^22^. This work’s first goal is to determine a material model suitable for an accurate representation of PDMS with a base to curing agent crosslinking ratio of 13:1 (or *n* = 0.08). PDMS in this particular blend shows the highest flexibility, elasticity and strain range without sacrificing its structural stability^9,23^. Starting from there, we then moved to fit the chosen material model for PDMS with slightly increased crosslinking ratio (increased *n*). A higher *n* value typically corresponds to higher Young’s Modulus (Fig. S2), consequently making the PDMS stiffer, tougher and less elastic. Our end goal is to provide a generalized equation within the chosen model to represent a wide range of PDMS blends.

The first part of the selection process involves multiple trial and error attempts to curve-fit experimental data of PDMS sample *n* = 0.08 shown in Fig. 2 into each hyperelastic model outlined in the previous section. The Levenberg–Marquardt algorithm was used to facilitate the non-linear least squares curve fitting process^24^. This solver was frequently used in similar studies involving hyperelastic materials, owing to its ease of use in the context of most finite element analysis software^25–27^.

**Figure 3:**
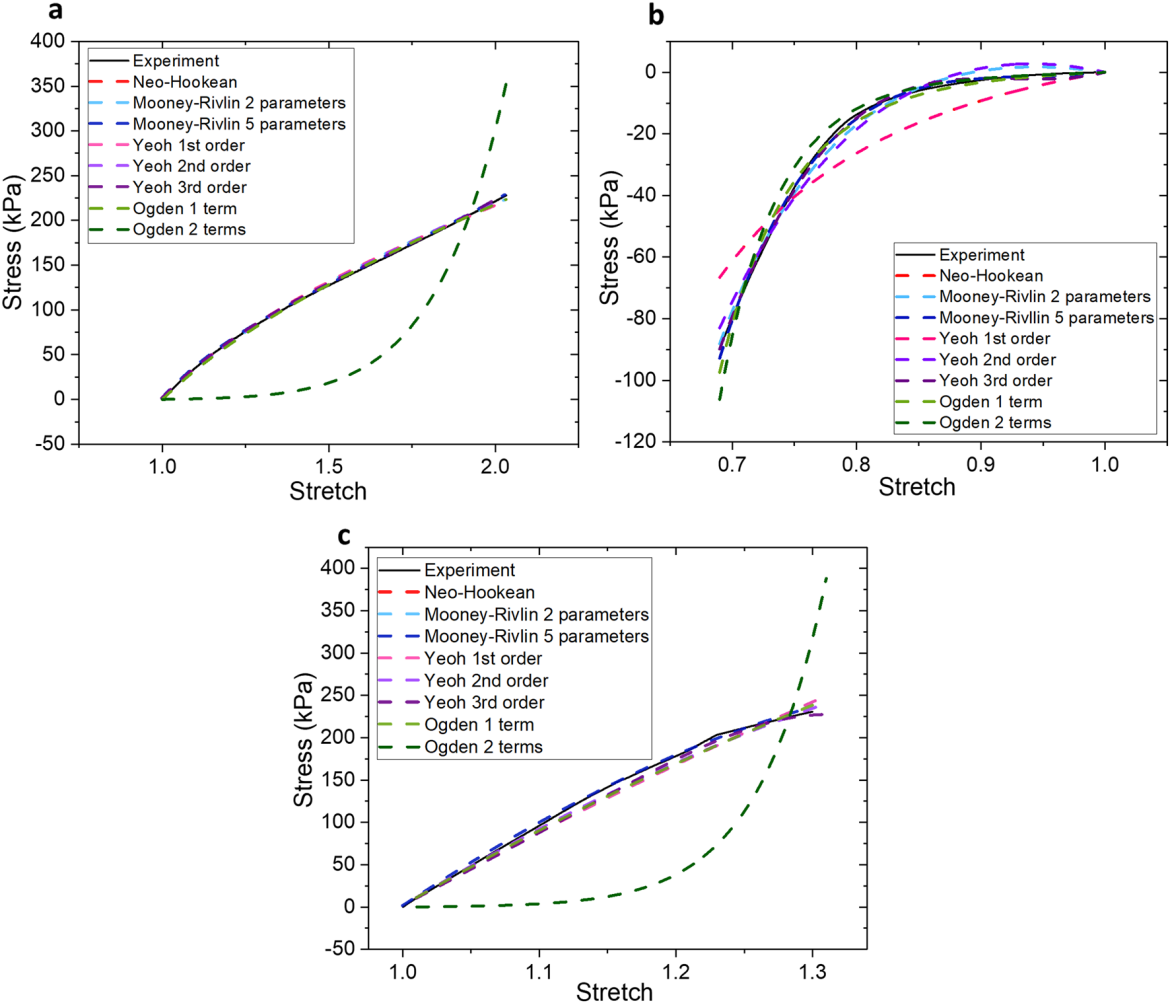
Least squares fit plots of PDMS *n* = 0.08 experimental data to Neo-Hookean, Mooney–Rivlin 2 parameters, Mooney–Rivlin 5 parameters, Yeoh 1st Order, Yeoh 2nd Order, Yeoh 3rd Order, Ogden 1 term, and Ogden 2 terms models for: (**a**) uniaxial tensile test; (**b**) uniaxial compressive test; (**c**) simple shear test.

Based on the curve-fitting results shown in Fig. 3, it is apparent that for tensile (Fig. 3a) and shear experiments (Fig. 3c), all models exhibit close fit with the experimental data, with the exception of Ogden 2 terms model. This massive discrepancy is possibly due to the nature of the formulation of Ogden model itself. The equations dictating Ogden model stipulate absolute independence from the use of deformation tensor invariants (*I*) and instead, rely on principal stretches, ${\lambda }_{i},$ where *i* = 1, 2, and 3^20^. This is a major difference separating the Ogden model from other models used in this paper. It is possible, however, to manipulate Ogden model into resembling another model by inserting certain numerical values into its parameters, ${\mu }_{p}$ and ${\alpha }_{p}$. Nevertheless, these parameters themselves hold no basis for physical interpretations, even with its highly accurate representation of most rubber and elastomer’s physical traits. Due to this, many parameter calculations done using Ogden model result in varying values. However, in some studies, it has been observed that in the tensile region, there is a tendency for curves to show strain softening at $\left|{\alpha }_{p}\right|<1$ and strain hardening at other ${\alpha }_{p}$ values^28^. This can be observed as well in our results in Fig. 3a, in which Ogden 1 term shows strain softening effect correlating well with our experimental data, while the curve for Ogden 2 terms exhibits strain hardening.

Interestingly, the same observation cannot be said for compression experiments, as depicted in Fig. 3b. As can be further clarified in Table 1, R^2^ coefficient of determination (which was used to determine the closeness and accuracy of the fit), shows that only 50% of the hyperelastic models tested are a close fit (above the level of 90%). This clearly illustrates the impracticability of many hyperelastic models for representing compressive deformations. While this can certainly be neglected in studies where tensile deformations are significantly prominent, in reality, Poisson’s effect dictates that even in tensile deformations, most structures will still undergo compression in a direction perpendicular to the tensile elongation. However, in the case of materials with negative Poisson’s effect, a different constitutive methodology should be performed instead.

**Table 1:** Goodness of fit (coefficient of determination, R^2^) values for all models.

Hyperelastic model	Mechanical test		
	Tensile	Compressive	Shear
Neo-Hookean	0.998393	0.855245	0.992391
Mooney–Rivlin 2 parameters	0.998704	0.990729	0.992391
Mooney–Rivlin 5 parameters	0.999988	0.998509	0.999139
Yeoh 1st order	0.998393	0.855245	0.992391
Yeoh 2nd order	0.999141	0.979324	0.995122
Yeoh 3rd order	0.999870	0.997810	0.998402
Ogden 1st order	0.998945	0.994170	0.994428
Ogden 2nd order	0.704377	0.977361	0.824771

A value closest to 1 shows best fit. See "Method“ section for R^2^ calculation description.

To proceed with the next series of investigation, we will need to select the hyperelastic model showing the highest accuracy to its experimental counterpart. Based on the calculated R^2^ coefficient of determination data listed in Table 1, this can be narrowed down to the Mooney–Rivlin 5 parameters model. Its calculated results prove to be the highest over the other models in all three mechanical experiments. Mooney–Rivlin models are known to be highly suitable for modeling small and medium strain applications, which are typically referred to 0–100% tensile force and up to 30% compression force^18,19^. These ranges fit well with PDMS’s natural stretch and compression tendencies. It is also worthy to note that Mooney–Rivlin models are incredibly versatile and allow accurate fit for not just basic tensile and compression mechanical tests, but for various shear experiments as well. While higher order modes of the model may improve the overall accuracy of the fit, the computational efforts required can be quite expensive. As such, we attempted to check the curve-fitting of Mooney–Rivlin 9 parameters using our experimental data and methodology, but the resulting plot (Supplementary Information 3) did not show significant change from the plot obtained from Mooney–Rivlin 5 parameters.

Finally, to conclude this investigation, the material parameters obtained for each hyperelastic model fitted are listed in Table 2. However, since we are only concerned with the model showing highest accuracy to the experimental data, we will only proceed with the parameters for the Mooney–Rivlin 5 parameters model.

**Table 2:** Material parameters for all models.

Hyperelastic Model	Material parameters (Pa)					
Neo-Hookean	μ					
	3.4933 × 10^5^					
Mooney–Rivlin 2 parameters	C_10_			C_01_		
	− 2.9481 × 10^5^			5.2082 × 10^5^		
Mooney–Rivlin 5 parameters	C_10_	C_20_	C_11_		C_01_	C_02_
	1311.9	19,515	− 9320.3		9491.6	14,683
Yeoh 1st order	C_1_					
	60,015					
Yeoh 2nd order	C_1_			C_2_		
	1.4697 × 10^5^			68,811		
Yeoh 3rd order	C_1_		C_2_		C_3_	
	1.5738 × 10^5^		1.4404 × 10^5^		41,951	
Ogden 1st order	$\mu 1$			α_1_		
	1.8803			0.8862		
Ogden 2nd order	μ1	α_1_		μ2		α_2_
	102.18	2.9583		102.18		2.9519

The experimental data fitted are a combination of all three mechanical tests (uniaxial tensile, uniaxial compressive, and simple shear).

### FEM simulations

**Figure 4:**
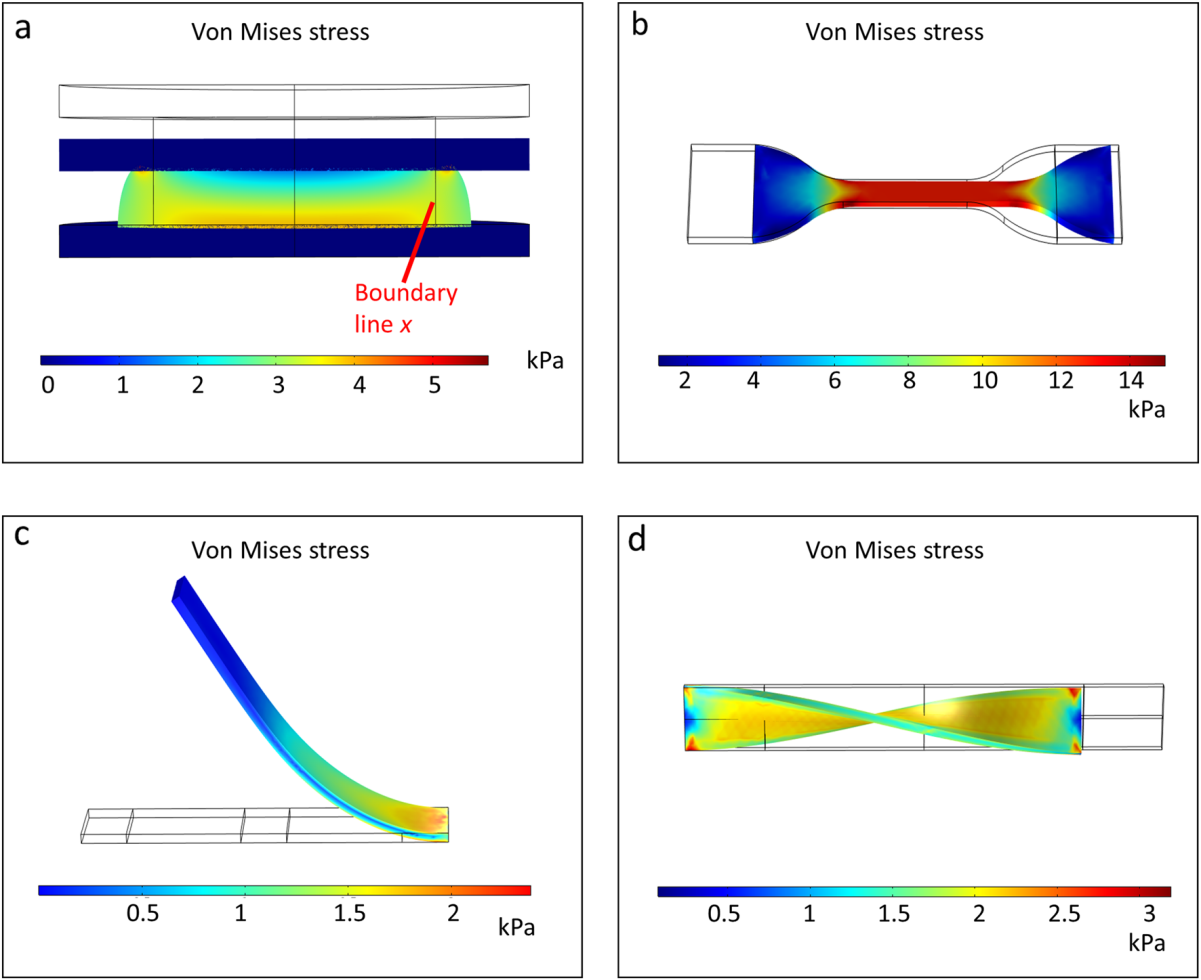
Finite element analysis showing von Mises plots of PDMS *n* = 0.08 modeled using the parameters defined by Mooney–Rivlin 5 parameters model. All simulations are representative of common mechanical deformations subjected to flexible, wearable devices: (**a**) normal compressive pressure; (**b**) uniaxial stretching; (**c**) unidirectional bending; (**d**) single-turn twisting.

To show the applicability of the Mooney–Rivlin 5 parameters model for modeling stretchable *n* = 0.08 PDMS, we prepared simulations demonstrating four basic mechanical deformations typical in wearable devices^24^. These mechanical deformations are; normal compressive pressure, uniaxial stretching, unidirectional bending, and single-turn twisting. The von Mises stress plots for the deformations are illustrated in Fig. 4.

In each simulation, one end of the domain was constrained, while the other end was subjected to varying levels of uniaxial displacement (pressure and stretching), unidirectional rotating frame (bending), and applied moment (twisting). All domains and boundaries are defined as PDMS using the Mooney–Rivlin 5 parameters model described in earlier section.

Figure 4a shows a cross sectional view of a 1 cm-thick cylindrical model of PDMS sandwiched between two steel plates. This PDMS-sandwich structure is a typical configuration for various flexible electronic devices such as capacitive pressure sensors and actuators. To mimic the structural deformation involved in such devices, a distributed compressive force was applied by forcing a 50% displacement of the top plate in the simulation. Subsequently, the von Mises stress contour is observed. The resulting stress distribution is gradually increased from top center to bottom. However, we see a decrease of stress instead, when moving radially outwards from boundary line *x*, where the PDMS starts to occupy a different volumetric space away from its original domain. One can also observe maximum stress value near the interface of PDMS and bottom plate, as well as the top edge directly outside boundary line *x*. These observations are similar to theoretically computed results in literature^29^.

In Fig. 4b, we can observe the von Mises plot of a dogbone-shaped PDMS structure corresponding to a uniaxial tensile stretch of about 15% strain (relative to the structure’s original dimension). A large portion of stress is focused on the narrower, middle boundary, where we can also observe stress uniformity. This correlates well with the ideal concept of the standard tensile test outlined by ASTM D412, which dictates that any structural analysis should be performed specifically within the middle boundary defined by the length called Gauge Length, GL (Supplementary Information 1). Another notable observation from the simulation is the thinning of the boundary layer within GL, as well as the slight necking near the curved edges at the top. These observations can likewise be observed in actual tensile stretch experiments shown previously in Fig. 2a.

Next, a slightly different approach was taken to simulate PDMS under bending. Instead of using the classic cantilever beam method, a rotating frame load was utilized instead, which was applied on one half of the length of a PDMS beam (Fig. 4c). Using this method, the observer is actually rotating together with the beam. As a consequence, all resulting stress distributions are calculated relative to the rotating half. The main reason for this setup was mainly because in wearable devices, deformations due to bending are typically not restricted to simple axial force applied to only one end of the structure, as is usually represented in the cantilever beam method. Instead, bending usually involves single or multidirectional rotations, arising from intrinsic movements from the skin, muscles, tendons, or bones. For example, a strip of tape adhered on the inner elbow will be deformed due to rotation of the forearm or the upper arm, and not by a single load application.

Using this method, the PDMS beam was bent with angular velocity magnitude of 15 rad/s in a time-dependent study of 0.1 s time step. This allows us to analyze the dynamic von Mises stress distribution within the strip as it is being continuously deformed. One such instances are recorded in Fig. 4c where we can observe the smooth progression of stress from the beam end where force is being applied, to the other fixed end. Naturally, we would see the highest stress concentration near the fixed end. It is also interesting to note that both the bottom and top surface of the strip contains higher stress value compared to its inner body, where the neutral axis lies.

To simulate twisting, a PDMS strip is subjected to an applied torsion moment of $3 \times 10^{ - 6} \;{\text{N}}\;{\text{m}}$ on one end. Although the other end is still kept fixed and constrained, both ends show symmetric stress distribution, as illustrated in Fig. 4d. Similar to bending deformation, one may also observe zero von Mises stress in its neutral axis.

These simulation results are meant to represent common mechanical deformations subjected to materials used in typical wearable electronics applications. As such, the structures involved are usually thin and flexible, with multiple load points or load gradients. Due to the complexity of such simulations, a number of restrictions and simplifications had to be done, including limiting the model to single layer structures, removing any factors that might cause statically indeterminate problems, simplifying effects of contact friction and adhesion, as well as allowing several discontinuities between fixed and moving boundaries.

### Effects of combining different mechanical experiments

As we increase the number of parameters or order of a model, the resulting output may become unstable or even produce non-physical solutions for more complicated simulations. To alleviate this problem, we attempted to provide different options or alternative solutions while still retaining the use of the Mooney–Rivlin 5 parameters model. This was carried out by investigating the effects of combining data from different mechanical experiments (tensile, compression, shear). Analysis of simulations performed using these different data combinations can then decide which combinations can provide results as accurate as the original plot.

In Table 3, we observe that the Mooney–Rivlin parameters obtained from the different combinations of mechanical experiments are rather varied from one another. However, this does not necessarily mean that the differences between simulations performed with these datasets are large. To ascertain this, more FEM simulations need to be performed.

**Table 3:** Material parameters for Mooney–Rivlin 5 parameters obtained for different combinations of mechanical deformation datasets.

Mechanical deformation	Material parameters (Pa)				
	C_01_	C_02_	C_10_	C_11_	C_20_
Tensile + comp + shear	9491.6	14,683	1311.9	− 9320.3	19,515
Tensile + comp	− 5713.9	15,702	10,969	− 3137.4	14,847
Comp + shear	1863.5	21,208	2353.9	− 6263.5	14,073
Tensile + shear	− 60,004	4646.7	1.167 × 10^5^	− 57,865	25,374
Tensile	31,432	− 106.44	36,124	− 491	3158.5
Compression	4424.6	18,291	− 336.38	− 4323.4	11,330
Shear	21,431	18,323	21,431	− 1.103 × 10^5^	18,323

**Figure 5:**
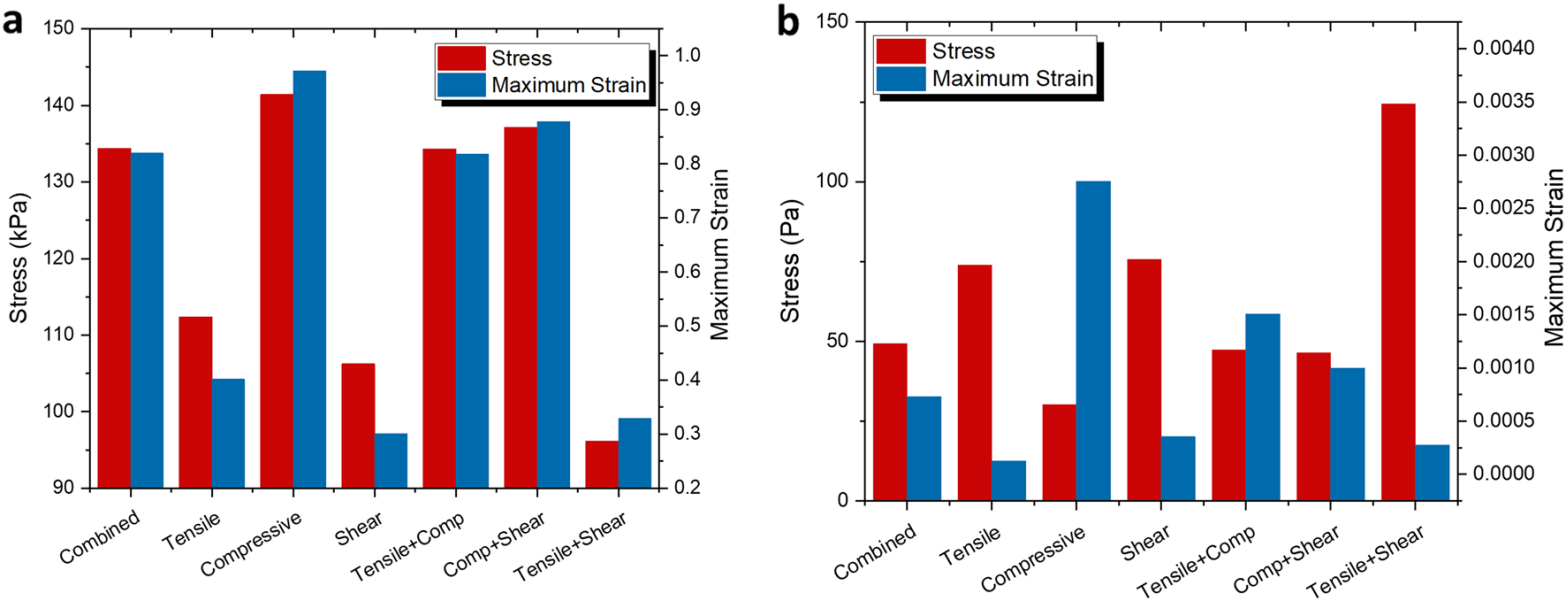
Von Mises stress and maximum strain plots simulated using different combinations of mechanical experiment datasets (combined, tensile, compressive, shear, tensile + compressive, compressive + shear, tensile + shear): (**a**) simulation of PDMS sample *n* = 0.08 undergoing uniaxial stretching; (**b**) simulation of PDMS sample *n* = 0.08 undergoing unidirectional bending.

It has been widely established that a higher number of experimental datasets would consequently produce a more stable, robust model^10,22^. Thus, the triple combination simulation (henceforth referred to as ‘Combined’) performed previously in Fig. 4 would stand to be the standard or point of reference to be compared to. In Fig. 5, two of the simulations (stretching and bending) were replicated using the data combinations in Table 3. Their von Mises stress and maximum strain values are then compared with the Combined values in Fig. 4.

Based on Fig. 5a, it is immediately apparent that the Tensile + Compression combination is remarkably accurate in both stress and maximum strain, making it the obvious choice for an alternative combination. The Compression + Shear combination follows closely after, with slightly higher values. One may still use this combination for any primary stretching simulations but a slight overshoot on the results should be expected. On the other hand, Tensile, Shear and Tensile + Shear should be disregarded as alternative choices due to their high deviations from the reference data.

For bending simulation of a rectangular beam, according to Fig. 5b, the closest result to the Combined values would be the Compression + Shear combination, followed by Tensile + Compression. However, for simulations that require higher accuracy, it is suggested to only use Combined datasets rather than any other alternative combinations. This is because bending deformations in general, are heavily governed by bending stress (tensile and compression stresses) and shear stress as outlined in the following equations:

13 $$ \sigma = \frac{Mc}{I} $$

14 $$ \tau = \frac{3}{2}\frac{V}{A} $$

where $\sigma$ and $\tau$ are bending stress and shear stress respectively, *M* is bending moment, *c* is the distance between the neutral axis and outer surface of the beam, *I* is the second moment of area, *V* is localized shear force, and *A* is the beam’s cross sectional area.

It is therefore advisable to include shear data when simulating bending deformations. This is also theoretically similar to twisting or torsion simulations, in which the relationship between torsion and shear stress, $\tau$, can be defined as:

15 $$ \tau = \frac{T}{{kwh^{2} }} $$

where *T* is applied torsion in ${\text{N}}\;{\text{mm}}$, and *k* is a constant defined by values of width, *w* and height, *h*, of the cross sectional beam.

Based on the overall result in Fig. 5, we may conclude that double combinations (two experimental data) show much higher accuracy in most cases. However, we may not necessarily need to disregard single datasets completely. Depending on the type of deformation and the resulting analysis measured, single datasets (Tensile, Compressive and Shear) can still be used as an alternative. An example of this is using Compressive data for measuring von Mises stress in a stretching simulation.

### Varying the base to curing agent blend of PDMS samples

**Figure 6:**
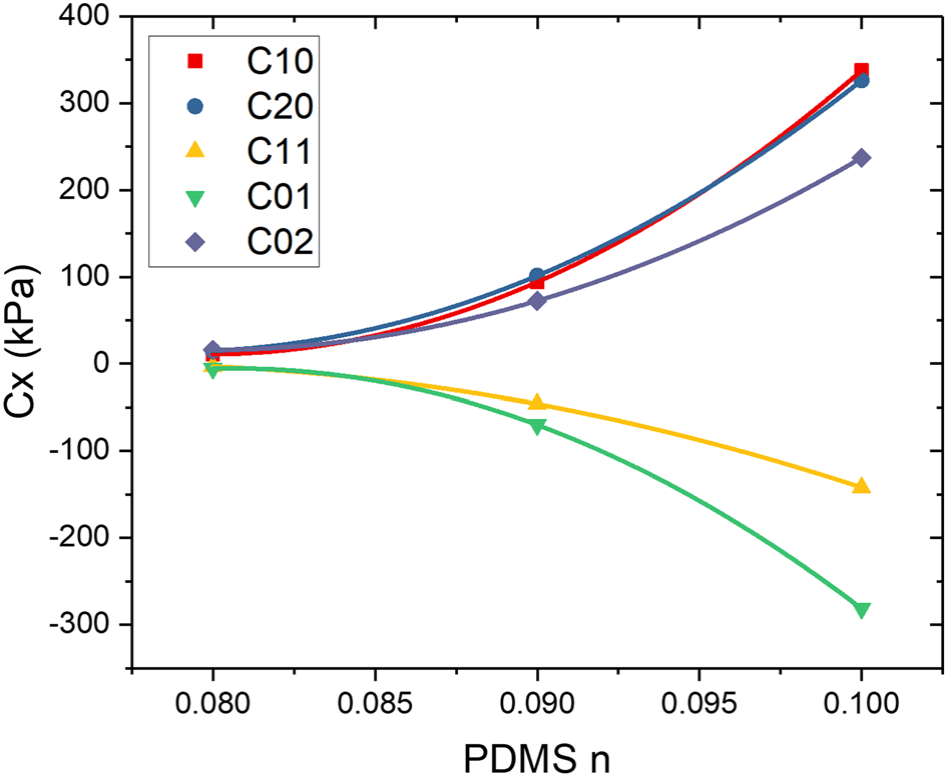
Parameter correlation plot showing material parameters for Mooney–Rivlin 5 parameter plotted for PDMS samples within the range of *n* = 0.08–0.1.

To show the applicability of Mooney–Rivlin 5 parameters model for wider range of PDMS concentrations, two more PDMS samples of *n* = 0.1 and *n* = 0.09 were investigated using similar modeling methodology. The resulting model parameters from the curve fitting process were plotted in Fig. 6. Using polynomial curve fitting method, we were then able to connect each parameter from all three PDMS samples to form a parameter correlation plot. This will then enable us to determine model parameters for other PDMS concentration or *n* without having to perform any additional experiments.

The correlation equations for all five parameters in the Mooney–Rivlin model are as follows;

16 $$ C_{10} = 5.12 \times 10^{3} - 1.28 \times 10^{5} n + 8.02 \times 10^{5} n^{2} $$

17 $$ C_{20} = 4.3 \times 10^{3} - 1.09 \times 10^{5} n + 6.92 \times 10^{5} n^{2} $$

18 $$ C_{11} = - 1.56 \times 10^{3} + 4.07 \times 10^{4} n - 2.65 \times 10^{5} n^{2} $$

19 $$ C_{01} = - 4.8 \times 10^{3} + 1.19 \times 10^{5} n - 7.37 \times 10^{5} n^{2} $$

20 $$ C_{02} = 3.44 \times 10^{3} - 8.6 \times 10^{4} n + 5.39 \times 10^{5} n^{2} $$

### Experimental validation

**Figure 7:**
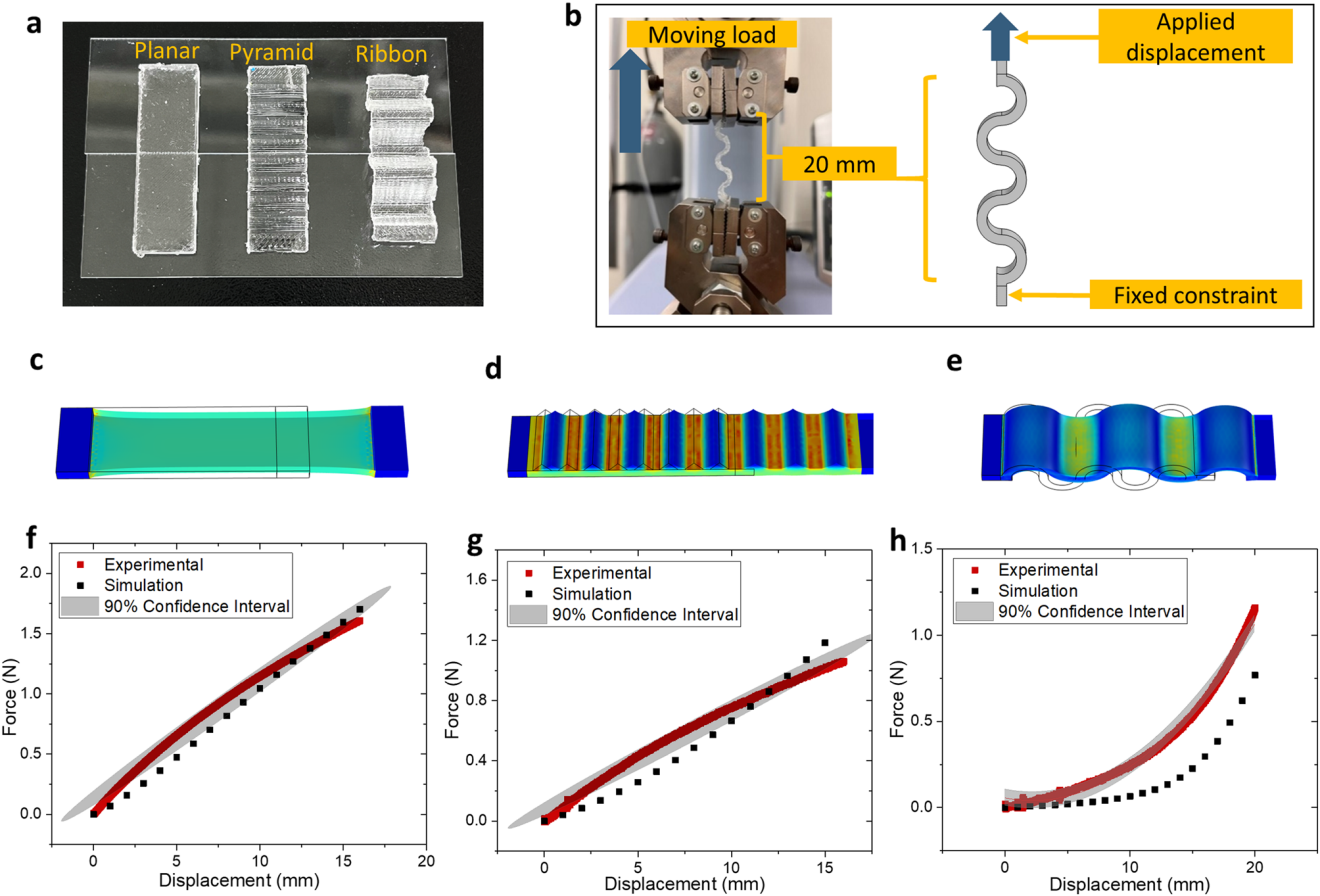
(**a, b**) Fabricated samples and experimental and simulation setup for validation experiment using PDMS *n* = 0.095: (**a**) planar, pyramidal microstructure and ribbon structure PDMS samples; (**b**) equipment setup and corresponding FEM simulation setup for uniaxial stretching of PDMS samples. (**c**–**e**) Simulation geometries corresponding to: (**c**) planar PDMS sample; (**d**) pyramidal microstructure PDMS sample; (**e**) ribbon PDMS sample. (**f**–**h**) Uniaxial force–displacement comparison between experimental and simulation results for (**f**) planar PDMS sample; (**g**) pyramidal microstructure PDMS sample; (**h**) ribbon PDMS sample.

Using the correlation plot, model parameters for PDMS *n* = 0.095 were extracted. Likewise, several samples of PDMS with the same *n* value were fabricated as illustrated in Fig. 7a. These samples vary in terms of their geometrical shape and structural design; a planar layer, a pyramidal microstructure layer, and a wave-like ribbon structure. Each sample was fabricated using 3D-printed molds and the CAD drawings used to design these molds were imported into COMSOL as illustrated in Fig. 7c–e. This ensures the absolute degree of similarity between the actual samples and the digital designs. The real samples were then stretched in a laboratory experiment (Fig. 7b) within range of 0–20 mm displacement. The force (N) required to make each stretch was recorded and the resulting force–displacement plots are shown in Fig. 7f–h, along with their 90% confidence interval. Within these figures as well, the simulated force from COMSOL Multiphysics using the same experimental setup were plotted to further show the comparison between real and simulated PDMS samples.

Both planar and pyramid samples show close and significant affinity between the experimental and simulated results. However, we believe that the simulations can be further improved with the addition of a suitable dampening factor such as viscoelasticity. In the case of the ribbon sample, while the higher range of the simulated plot does not strictly lie within the confidence interval region of the experimental result, the trend and shape of the curve is distinctively similar.

As mentioned earlier, adding in viscoelastic attributes would potentially improve the simulation accuracy, particularly in defining the nonlinear shape of the curve. PDMS itself is well known for its highly exploitable viscoelastic effects, and many FEM software including COMSOL do provide sufficient integration with viscoelasticity. In addition, combining viscoelastic expressions will allow us to conduct more time-specific, transient simulation which could be essential in wearable electronics and bio-diagnostic devices. In the future, we endeavor to continue this study within this framework.

## Methods

### Fabrication of PDMS samples

In order to fabricate the PDMS samples for stress–strain characterization plots, a blend of base to curing agent (Sylgard 184, Dow Corning) was prepared according to variable *n*, introduced in earlier section, followed by a 45 min degassing process to remove air bubbles. The PDMS was then poured into 3D-printed (FlashForge Finder) molds designed according to standards specified for uniaxial tensile test, uniaxial compressive test and planar shear test (ASTM D412 (Type C), ASTM D575, and ASTM D1002 (single lap shear test) respectively). The solution was then left overnight at room temperature to allow full curing.

For the experimental validation section of this work, the PDMS samples were fabricated via 3D-printed molds, using polylactic acid (PLA) as the molds’ core material. For this purpose, several molds were created. The first, was a planar mold composed of a flat rectangular structure with a hollow 25 mm × 10 mm × 3 mm feature etched in the middle. Likewise, to fabricate PDMS microstructure validation samples, similar molds with reduced thickness (1.5 mm) were fabricated but with additional pyramidal shaped lines (base to height distance: 1.5 mm) running parallel to the shorter sides of the mold. The third microstructure sample is a unique design replicating the shape of a symmetrical ribbon or wrinkled structure.

### Characterizations of PDMS layers

All mechanical tests were performed via the Universal Testing Machine or UTM (Shimadzu EZ-LX) at room temperature. Tensile tests were carried out by clamping both ends of the dogbone PDMS samples (based on ASTM D412 (Type C)) within the UTM and applying stretching force at a rate of 20 mm/min until fracture occurred (Fig. 2a). On the other hand, compression experiments were done by sandwiching ASTM D575 PDMS samples between two metal plates and applying 1 mm/min compressive force until 30% nominal strain was achieved (Fig. 2b). A sandpaper was placed in between the samples and metal plates to increase friction and adhesion, and each sample underwent 3 compression series for optimum accuracy. Finally, for simple shear tests, the samples based on ASTM D1002 standards were attached in between two metal strips using commercial liquid adhesive (Loctite 401) as shown in Fig. 2c. The metal strips were cleaned with acetone and sandpaper prior to attachment to remove contaminants. Each free end of the strip was then clamped on UTM’s grippers, and tensile stress was applied at rate of 1.3 mm/min until fracture due to adhesive failure occurred.

### Calculation of coefficient of determination (R^2^)

To check for the goodness of fit of the hyperelastic models to the experimental data, a statistical method called the coefficient of determination, or R^2^ was used. This method essentially defines the strength of a relationship between two variables, which in this case would be the experimental data and the fitted hyperelastic model.

The result will fall within a range of 0–1, where 0 denotes poor fit, and 1 the best possible fit. A value of 1, for instance, means that all of the data points of the experimental data fall within the results of the line formed by the curve fitted hyperelastic model. This can also determine the likelihood of future events falling within the predicted outcomes, for example if more samples are added.

The equation for R^2^ used in this study is as follows;

$$ {\text{R}}^{2} = 1 - \frac{RSS}{{TSS}} $$

where *RSS* is the sum of squares of residuals, and *TSS* is total sum of squares.

### Supplementary Information

Supplementary Information.

## Data Availability

The raw data and processed data required to reproduce these findings are available in the Mendeley Data repository, https://data.mendeley.com/datasets/mwmzpgrzs3.
