# Supplementary material for: Comprehensive constitutive modeling and analysis of multi-elastic polydimethylsiloxane (PDMS) for wearable device simulations
Source: Sci Rep. 2023 Oct 27;13:18413. doi: 10.1038/s41598-023-45372-0 (PMC10611765; doi:10.1038/s41598-023-45372-0)
Supplement: Supplementary file 1 — Supplementary Information. [file 41598_2023_45372_MOESM1_ESM.docx]

Supplementary Information

*Nora Asyikin Binti Zulkifli, Geon Dae Moon, Dong Choon Hyun and Sungwon Lee*

**Supplementary Information 1:** Dimensions for all polydimethylsiloxane (PDMS) samples prepared for mechanical deformation experiments.


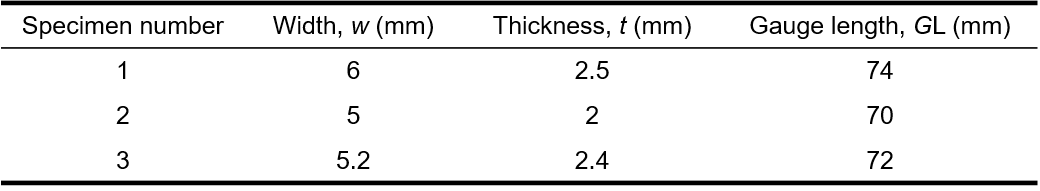
**Supplementary table 1**: Actual dimensions for each tensile (uniaxial) test sample.


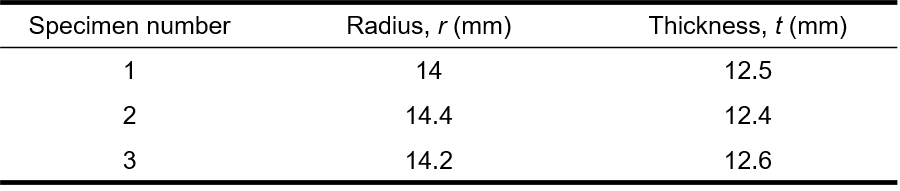


**Supplementary table 2**: Actual dimensions for each compression (uniaxial) test sample.


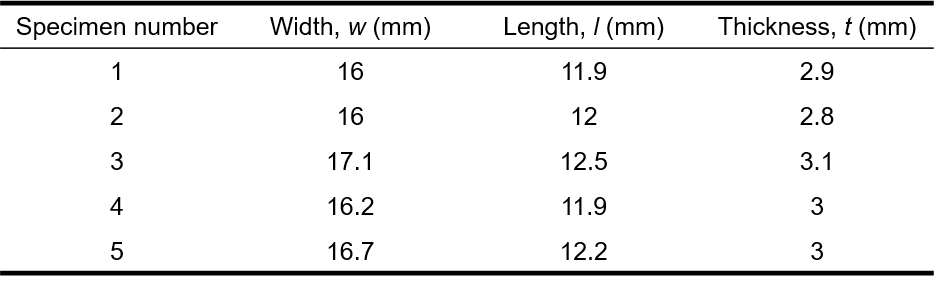


**Supplementary table 3**: Actual dimensions for each shear (planar) test sample


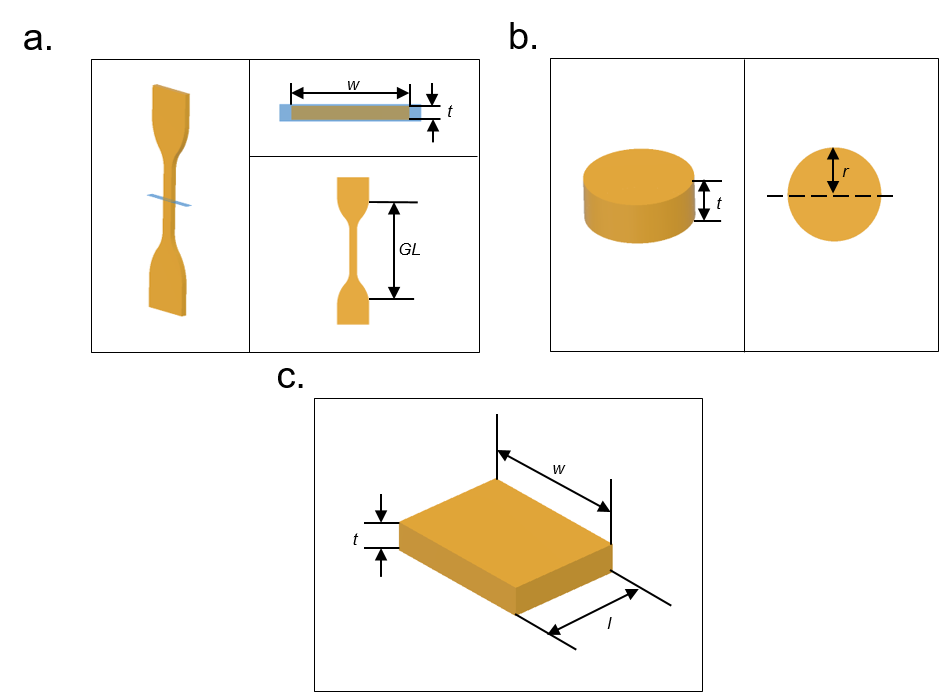


**Figure S1.** PDMS sample dimensions for mechanical deformation experiments: a) uniaxial tensile test (ASTM D412 (Type C)); b) uniaxial compressive test (ASTM D575); c) simple shear test (ASTM D1002).

**Supplementary Information 2:** Experimental data showing nominal stress-strain plots of PDMS samples fabricated with different blending ratio, *n*.


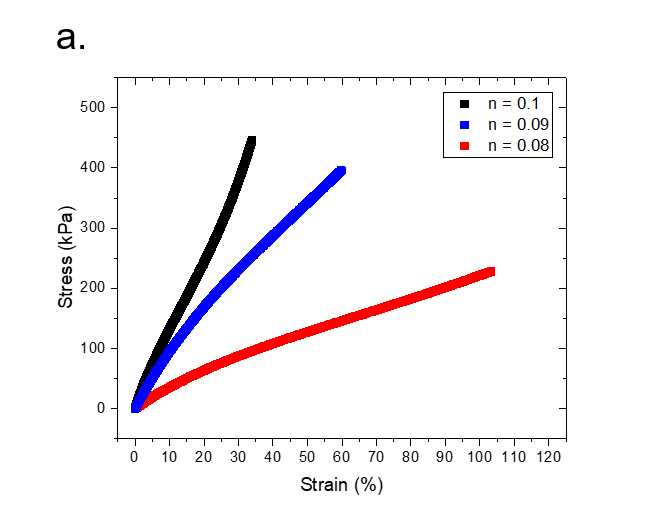


**Figure S2.** Stress-strain plot of PDMS fabricated with varying blending ratio, *n.*

**Supplementary Information 3:** Strain energy density function, *W* equation for Mooney-Rivlin 9 parameters model, its corresponding curve-fitted plot, and comparison to fitted plots for Mooney-Rivlin 5 parameters model.

$$\boldsymbol{W}=\boldsymbol{C}_{10}\left( \boldsymbol{I}_{1}-3 \right)+\boldsymbol{C}_{01}\left( \boldsymbol{I}_{2}-3 \right)+\boldsymbol{C}_{20}\left( \boldsymbol{I}_{1}-3 \right)^{2}{+\boldsymbol{C}}_{11}\left( \boldsymbol{I}_{1}-3 \right)\left( \boldsymbol{I}_{2}-3 \right)+\boldsymbol{C}_{02}\left( \boldsymbol{I}_{2}-3 \right)^{2}+\boldsymbol{C}_{30}\left( \boldsymbol{I}_{1}-3 \right)^{3}+\boldsymbol{C}_{21}\left( \boldsymbol{I}_{1}-3 \right)^{2}\left( \boldsymbol{I}_{2}-3 \right)+\boldsymbol{C}_{12}\left( \boldsymbol{I}_{2}-3 \right)^{2}\left( \boldsymbol{I}_{1}-3 \right)+\boldsymbol{C}_{03}\left( \boldsymbol{I}_{2}-3 \right)^{3}+\frac{1}{\boldsymbol{D}}\left( \boldsymbol{J}-1 \right)^{2}$$

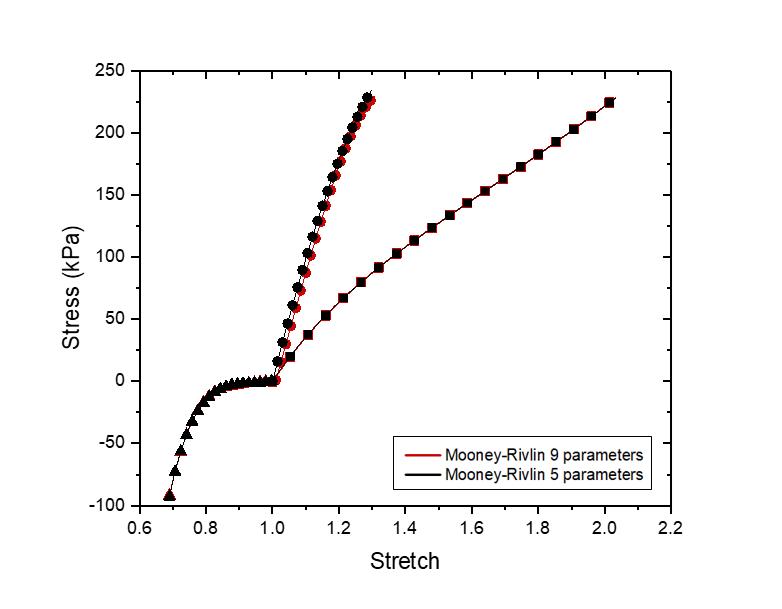


**Figure S3.** Comparison between curve-fitted plot of Mooney-Rivlin 9 parameters and curve-fitted plot of Mooney-Rivlin 5 parameters
